# Pediatric trauma over a decade: demographics, mechanisms of injury, and mortality at a major Danish trauma center—a retrospective cohort study

**DOI:** 10.1186/s13049-025-01348-9

**Published:** 2025-03-10

**Authors:** Mette Schytt Price, Nikolaj Raaber, Per Hviid Gundtoft, Frederik Trier

**Affiliations:** 1 Research Center for Emergency Medicine, Aarhus University, Aarhus, Denmark; 2 Department of Clinical Medicine and Emergency Department, Aarhus University Hospital, Aarhus, Denmark; 3 Department of Orthopedic, Aarhus University Hospital, Aarhus, Denmark; 4 Department of Anesthesiology and Intensive Care, Gødstrup Hospital, Gødstrup, Denmark; 5 Department of Emergency Medicine, Horsens Regional Hospital, Horsens, Denmark

**Keywords:** Pediatric trauma, Trauma team activation, Age-specific prevention, Trauma epidemiology, Injury severity score, Mechanism of injury

## Abstract

**Background:**

In recent decades, the number of fatal accidents among children and adolescents has declined. Nevertheless, trauma remains a significant cause of death among children and adolescents in high-income countries, despite significant advancements in prevention and care. Pediatric trauma patients differ substantially from adults in terms of their physiology, anatomy, and daily activities; therefore, they show distinct injury patterns and require different care. The aim of this study was to investigate mortality from trauma in pediatric patients admitted by trauma team activation at the Aarhus University Hospital Trauma Center (AUH-TC) in a highly developed country with exceptionally low child mortality, where trauma is a leading cause of death in this age-group. By evaluating trends in demographics, mechanisms of injury, injury severity, and outcomes, this study aims to provide insights into trauma care and outcomes.

**Methods:**

This retrospective cohort study included 1,037 pediatric patients (< 18 years old) consecutively admitted by trauma team activation from 1 January 2011 to 31 December 2021. The pediatric patients accounted for 14% of the total trauma population, which consisted of 7307 patients in total. Data on demographics, Injury Severity Score (ISS), mechanism of injury, and 30-day mortality were analyzed. Descriptive statistics were reported.

**Results:**

Boys accounted for 58% of the patients (n = 595). Falls were the mechanism of injury in 47% (n = 308) of children under 13 years old, while traffic-related injuries accounted for 38% (n = 139) of adolescents aged 14–17 years. Severe injuries (ISS > 15) were associated with traffic accidents in 25% of cases. The number of traumas peaked on weekends (71%) and during the spring/summer (29%). The ISS was greater than 15 in 13% (n = 130) of the patients, and the overall 30-day mortality rate was 1.6% (n = 17).

**Conclusions:**

This study found no significant change in pediatric trauma incidence at AUH-TC over a decade. In Denmark, the few children with an ISS above 15 are predominantly injured in traffic accidents, with risk increasing with age. There was a low incidence of patients with an ISS above 15, and mortality rates were lower than in similar studies. These findings on injury patterns and severity may aid in risk assessment, accident prevention, and hospital resource planning. Further research with extended follow-up is recommended to assess potential trends in trauma mechanisms over time.

## Background

Trauma remains the leading cause of death in children and adolescents in high-income countries, despite significant advancements in prevention and care including prehospital management, surgical interventions, critical care in pediatric intensive care units, and rehabilitation programs [1–4]. One explanation for this is that the patterns of pediatric trauma have shifted due to changing behavioral patterns among children and adolescents, including less active lifestyle and reduced unsupervised outdoor play [1, 5, 6]. These shifts highlight the importance of reassessing the mechanisms and outcomes of pediatric trauma patients to ensure that care protocols remain relevant and effective.

Pediatric trauma patients differ substantially from adults in terms of their physiology, anatomy, and daily activities; therefore, they also differ in their injury patterns and care needs [7].

Nesje et al. conducted a 13-year study on pediatric trauma in Norway, analyzing 873 patients aged 0–17 admitted to a trauma center in a setup very similar to Danish setups. The study found that traffic accidents (61%) and falls (27%) were the most common MOI. Severe injuries (ISS > 15) were observed in 15% of patients, with a 30-day mortality rate of 1%. Their findings highlighted a low incidence of severe injuries and mortality in a developed healthcare system, with limited need for intensive care and emergency surgery [8].

A study involving 24,218 pediatric trauma patients from the UK highlighted that head trauma was most common in infants, while limb and thoracic injuries were prevalent in older children [9]. Furthermore, patients with low Glasgow Coma Scale (GCS) scores showed significantly increased mortality [9]. Similarly, a study in Germany on 517 children receiving trauma care between 1997 and 2003 revealed traffic-related accidents to be the leading cause of pediatric trauma, with mortality rates varying by age group [10]. However, research focusing on recent trends in Scandinavia is limited and this study aims to investigate trends, mechanism of injury, injury severity and 30-day mortality.

In Denmark, pediatric trauma accounts for approximately 20% of all trauma cases [11], yet only a few studies have investigated the mechanism of injury (MOI), the Injury Severity Score (ISS), and 30-day mortality in children [12–14]. We hypothesized that over a ten-year study period, demographic changes and shifts in children's daily activities will be reflected in the types and severity of trauma injuries. The aim of this study was to investigate mortality following trauma in pediatric patients admitted by trauma team activation at the Aarhus University Hospital Trauma Center (AUH-TC) in a highly developed country with exceptionally low child mortality, where trauma-related deaths constitute one of the leading causes of mortality in this age-group. By evaluating trends in demographics, mechanisms of injury, injury severity, and 30-day mortality among children and adolescents (aged 0–17 years), this study aims to provide insights into trauma care and outcomes.

## Methods

This study was a ten-year retrospective cohort study compiled at the second-largest trauma center in Denmark from 1 January 2011 to 31 December 2021. Data were collected prospectively through the local Trauma Registry of AUH-TC. The study was conducted and reported according to the STROBE guidelines [15].

### Inclusion and exclusion

We included all patients under 18 years, alive on admission, and registered in the trauma registry. Patients with injuries from drowning, burn injuries, and poisoning are not registered in the trauma database and were therefore not included. Furthermore, patients initially admitted to other hospitals in the region and subsequently transferred to the regional trauma center, fulfilling the inclusion criteria, were included. The category “Violence” includes incidents such as physical assault, self-harm, and attacks while the category “Other” encompasses injuries related to animals, entrapment, crush injuries, sports injuries, and machinery-related injuries, among others.

### Setting

The catchment area for pediatric trauma patients with minor injuries as isolated factures covers the municipality of Aarhus, which has a population of 36,000 of whom 65,000 are under the age of 18 years.

The study was conducted in the Central Denmark Region, which has a well-developed healthcare infrastructure comparable to the rest of Denmark. No location within the region is more than 60 km away from one of the five acute care hospitals. Additionally, the Helicopter Emergency Medical Service (HEMS) ensures that Aarhus University Hospital can be reached within 60 min, regardless of where an injury occurs within the region [16]. The geography of the region is relatively uniform, with flat terrain and excellent infrastructure, including well-maintained roads. While Aarhus is the largest city, the surrounding areas consist primarily of smaller towns with populations ranging from 10,000 to 50,000.

### Trauma system and regional healthcare structure

AUH-TC receives major trauma patients from Central Denmark Region covering a population of 1.3 million of which 275,000 are under the age of 18 years [4]. In addition to AUH-TC, four acute hospitals service the Central Denmark region. In the Danish trauma system, "acute hospitals" are general hospitals capable of handling a wide range of medical emergencies, including minor injuries, but they lack some medical specialties. Key specialties such as neurosurgery, cardiovascular surgery, and thoracic surgery are only available at trauma centers, which are highly specialized facilities that offer comprehensive care for severe or life-threatening injuries through multidisciplinary teams and advanced equipment [17].

### Trauma criteria and injury severity scoring

The TTA criteria in the Central Denmark region are based on an algorithm established at the regional level (Appendix 1). Danish trauma centers utilize the Abbreviated Injury Scale (AIS) [18] to assess trauma patients' injuries. The AIS© 2005 Update 2008 manual was used for scoring the patients. The AIS score describes injuries within anatomical regions as well as injury severity ranging from 1 to 6, six being the most severe [19]. The ISS [18] is then used to assess the severity of the trauma and is calculated by summing the square roots of the highest AIS values from the three most affected regions. In the present study, ISS values were calculated by AIS-authorized physicians or medical students. Severely injured patients were defined by an ISS > 15 [20].

Due to restructuring, the registry only contains AIS scores from January 1st 2014 to December 31st 2021, whereas ISS scores are available for the entire study period.

### Statistical analysis

The incidence rate was calculated using the population count for persons under the age of 18 years in the Central Denmark region. The population count in the Central Denmark Region was collected retrospectively using the publicly available Statistics Denmark website [4]. Data for 30-day mortality were gathered through the electronic health record system using the Danish civil registration number. This system contains vital status for citizens who reside in Denmark. Descriptive statistics were presented as numbers and percentages. Continuous data were reported as means and standard deviations, whereas data lacking a normal distribution were reported as medians and interquartile ranges (IQRs). STATA version STATA/IC 18.0 (StataCorp. 2024. Texas, USA) was used for all statistical analyses.

### Ethics

This was a registry study and was therefore exempted from trial registration and patient informed consent in accordance with Danish law, primarily because of the anonymity of the data. The Trauma Registry of AUH-TC and the study were approved by the Central Denmark Region (1-45-70-71-23). Indirect identification is theoretically possible. However, the data are presented in an aggregated format to minimize this risk in the publication.

## Results

A total of 1037 children and adolescents aged < 18 years were included during the ten-year study period. The average annual number of pediatric trauma patients was 94. Overall, 58% of the patients were boys. Boys accounted for more than half of all age groups, with their percentage increasing from 53% in the youngest group to 62% in the oldest group.

### Time and season

In general, most accidents (93%) occurred between 08:00 and 23:59. In the 14–17 year age group, most accidents occurred between 16:00 and 23:59 (54%), and approximately four times as many accidents occurred at night between 00:00 and 07:59 (14%) compared to the remaining age groups.

Most accidents happened over the weekend (Saturday and Sunday) across all age groups (71%), with the 9–13 year age group particularly prone to accidents, accounting for 78% compared to weekdays (Monday to Friday).

Fewer accidents occurred from December to February (17%) across all age groups compared to the rest of the year, divided into seasons (28%) (Table 1).

**Table 1:** Patient characteristics and time of injury stratified by age groups

Age groups	0–4 years, n (%)	5–8 years, n (%)	9–13 years, n (%)	14–17 years, n (%)	Total, n (%)
*Gender*					
Girl	115 (47)	70 (43)	112 (44)	141 (38)	438 (42)
Boy	131 (53)	93 (57)	142 (56)	229 (62)	595 (58)
*Time of arrival*					
00:00 to 07:59	8 (3)	6 (4)	7 (3)	50 (14)	71 (7)
08:00 to 15:59	123 (50)	70 (43)	120 (48)	119 (32)	432 (42)
16:00 to 23:59	114 (47)	87 (53)	124 (49)	200 (54)	525 (51)
*Day of arrival*					
Weekday (Monday to Friday)	80 (33)	45 (28)	56 (22)	118 (32)	299 (29)
Weekend (Saturday and Sunday)	116 (67)	118 (72)	200 (78)	254 (68)	738 (71)
*Season of injury*					
December to February	39 (16)	24 (15)	47 (18)	70 (18)	180 (17)
March to May	71 (29)	59 (36)	75 (29)	99 (27)	304 (29)
June to August	79 (32)	45 (28)	68 (27)	103 (28)	295 (29)
September to November	57 (23)	35 (21)	66 (26)	100 (27)	258 (25)

### Age-groups

As shown in Table 2, the MOI varied across the different age groups. Overall, fall-related injuries were the most common MOI in children up to age 13: 0–4 years (63.8%), 5–9 years (46.3%), 9–13 years (30.7%), and 14–17 years (12.7%). Among patients aged 14–17 years, traffic-related accidents were the predominant MOI (38%). Bicycle injuries were more frequent in the 9–13 year age group (21%) compared to the rest of the groups: 0–4 years (3%), 5–9 years (12%), and 14–17 years (13%).

**Table 2:** Total number of MOI, ISS, and 30-day mortality stratified by age groups

Age groups	0–4 years, n (%)	5–8 years, n (%)	9–13 years, n (%)	14–17 years, n (%)	Total, n (%)
*Mechanism of injury (MOI)*					
Traffic	31 (12.8)	35 (21.6)	54 (21.3)	139 (37.7)	259 (25.2)
Bicycle	8 (3.3)	19 (11.7)	54 (21.3)	48 (13.0)	129 (12.6)
Pedestrian	14 (5.8)	15 (9.3)	15 (5.9)	37 (10.0)	81 (7.9)
Fall	155 (63.8)	75 (46.3)	78 (30.7)	47 (12.7)	355 (34.5)
Horse	7 (2.9)	1–5 (1.2)	24 (9.5)	47 (12.7)	80 (7.8)
Violence	1–5 (0.4)	0 (0.0)	1–5 (0.8)	24 (6.5)	27 (2.6)
Other	27 (11.1)	16 (9.9)	27 (10.6)	27 (7.3)	97 (9.4)
*Injury severity score (ISS)*					
ISS, median (IQR)	4 (1 to 6)	4 (1 to 9)	4 (1 to 8)	5 (1 to 10)	4 (1 to 9)
ISS 1–6: mild	185 (75.2)	106 (65.0)	187 (73.1)	220 (59.1)	698 (67.3)
ISS 7–15: moderate	42 (17.1)	36 (22.1)	45 (17.6)	86 (23.1)	209 (20.2)
ISS > 15: severe	19 (7.7)	21 (12.9)	24 (9.4)	66 (17.7)	130 (12.5)
*Mortality*					
30-day mortality	1–5 (1.6)	1–5 (1.8)	1–5 (1.6)	6 (1.6)	17 (1.6)
30-day survival	242 (98.4)	160 (98.2)	252 (98.4)	366 (98.4)	1020 (98.4)
30-day mortality, ISS > 15	1–5 (16)	1–5 (14)	1–5 (17)	5 (8)	15 (12)

### Injury severity score, mechanism of injury and 30-day mortality

The median ISS was 4 (range 1–75). Of the 1,037 patients, 130 (13%) had an ISS > 15. The proportion of patients with an ISS above 15 increased with age, peaking at 18% in the 14–17 year age group.

The overall 30-day mortality rate was 1.6%. Among patients with an ISS > 15, the 30-day mortality rate was 12%, indicating a substantially higher risk of death in this group compared to those with less severe injuries. In total, 12% of the patients with ISS > 15 did not survive beyond 30 days.

**Fig. 1:**
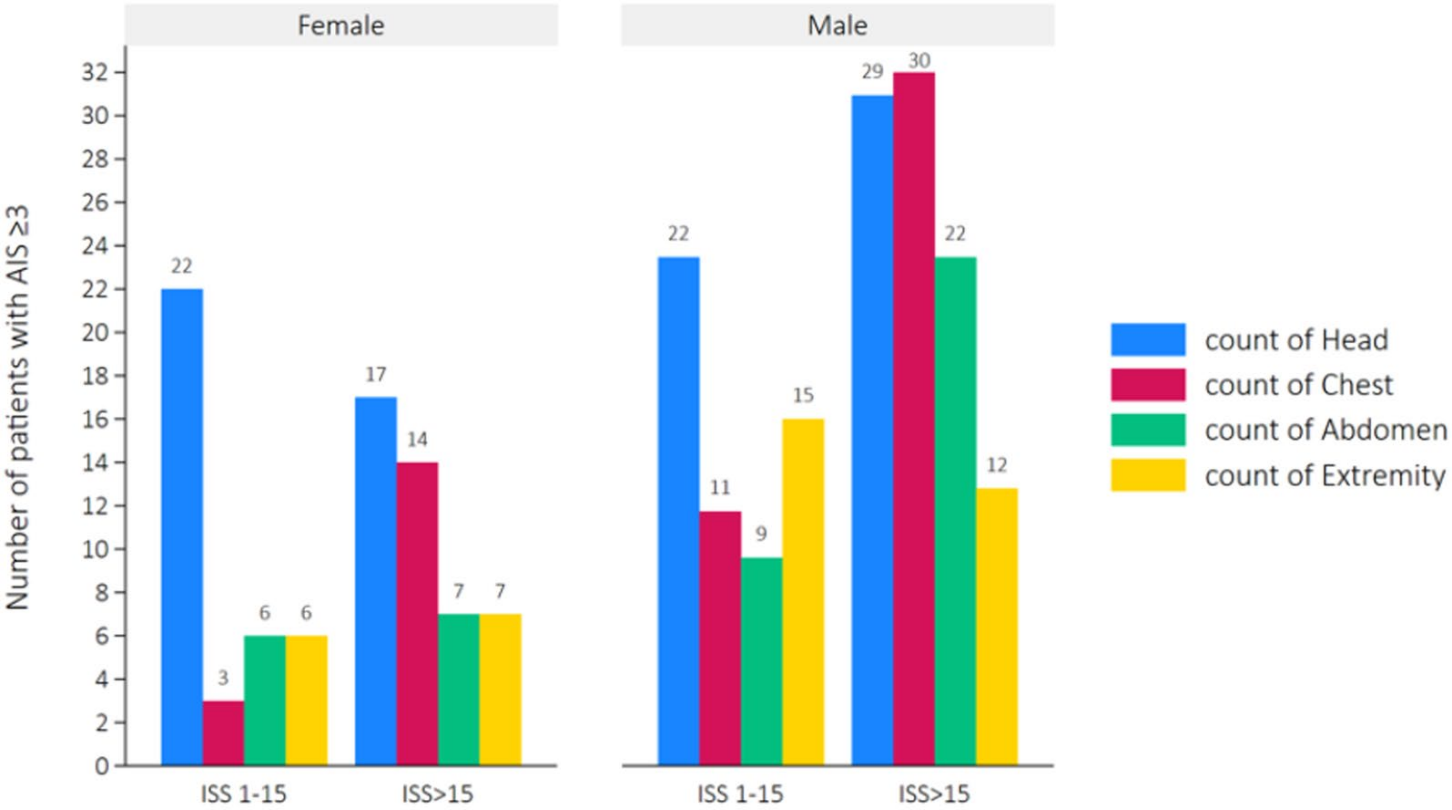
Number of patients with isolated injuries by body region, ISS (1–15 vs. 16–75), and gender

Head and thoracic traumas were most frequent among patients with ISS > 15. Boys with ISS > 15 (Fig. 1) more often sustained head (n = 29), thorax (n = 30), and abdominal (n = 22) injuries compared to boys with ISS < 15, who mostly had head (n = 22) and extremity injuries (n = 15). For girls, thoracic injuries were more common in girls with ISS > 15 (n = 14) than in girls with ISS < 15 (n = 3), while head injuries were more common in girls with ISS < 15 (n = 22).

### Trends in trauma mechanism

**Fig. 2:**
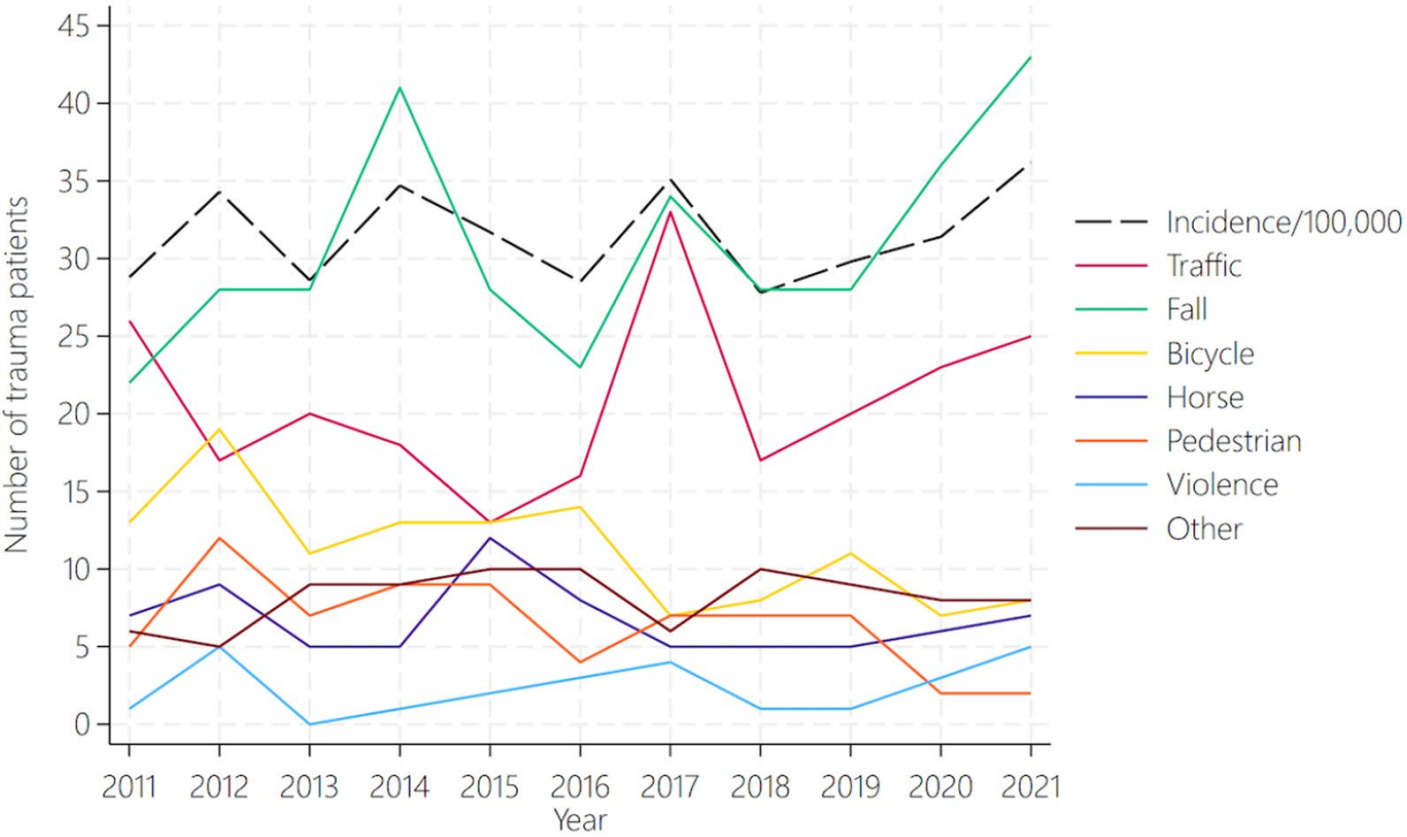
Trends in pediatric trauma mechanisms (number of traumas) and incidence (number of trauma patients per 100,000 inhabitants) in the Central Denmark Region from 2011 to 2021

The MOI varied over the decade but with no distinct trend (Fig. 2). The incidence rates varied between years, with notable peaks of 35.1 per 100,000 inhabitants in 2017 (95% CI 28.4–42.8) and 36.2 per 100,000 inhabitants in 2021 (95% CI 29.4–44.1).

A slight upward trend in incidence, fall injuries and traffic-related injuries was seen. Traffic-related injuries showed the most marked increase, particularly after 2016, whereas falls fluctuated, peaking in 2013 and 2016 before stabilizing. Bicycle-, pedestrian-, and violence-related traumas remained consistently low.

## Discussion

This study used data from the AUH-TC Trauma Registry to describe the characteristics, time trend, MOI, ISS, and 30-day mortality of pediatric trauma patients received by the trauma team at Denmark's second major trauma center in the decade from 2011 to 2021.

The age and gender distribution resembled those reported in previous studies from Denmark [8, 12–14, 21].

The dominating type of injury was caused by blunt mechanisms. Falls were the most common MOI in younger children (35%), whereas traffic-related accidents were more frequent in adolescents (aged 14–17 years) (25%). Traffic-related injuries most often resulted in an ISS > 15, and affected 17% of the boys and 18% of the girls. By comparison, falls led to an ISS > 15 in 12% of the boys and 10% of the girls. This shift in MOI with age has been well documented in previous studies [8, 12, 22].

The proportion of traffic-related injuries in our study, at 25%, was lower than the proportions reported in most previous studies [12, 14, 23]. For example, Beck et al. [24] reported that 43% of pediatric traumas were traffic related, while Faergeman [13] reported a proportion of 56%. Both studies considered pediatric trauma patients with ISS > 15, which could explain the discrepancy. Similarly, studies from Sweden, Germany, and Austria have reported higher rates of traffic-related injuries, ranging from 64 to 81% [25–27]. Studies from Canada and the United States have found that traffic-related injuries comprise between 31 and 39% of pediatric trauma cases [22, 28].

Most of the patients in our study had a low ISS score < 15 (87%). The median ISS score was 4, and 12% of the patients had an ISS > 15. The overall 30-day mortality rate of all pediatric trauma patients was 1.6%. These findings differ from a similar study from Denmark that examined a comparable study population but reported an ISS > 15 in 16% of their patients and a mortality rate of 5.2% [12].

One explanation for this difference could be that the threshold for trauma calls at our hospital is very low. As a result, we see a high proportion of children with injuries from falls, although these rarely result in a high ISS. This leads us to question whether we are utilizing our resources appropriately. The observed transition from falls to traffic accidents as children grow older also highlights the importance of age-specific prevention strategies, with a particular focus on enhancing road safety measures for adolescents.

The annual number of severely injured children was low, indicating that only a limited number of physicians gain clinical experience with this patient group. Moreover, many of the patients with an ISS > 15 were 15 years or older, and their physiology closely resembles that of adults. This reduces the necessity for dedicated pediatric expertise. Consequently, implementing pediatric trauma teams, as seen in the USA, does not appear to be a cost-effective solution for our hospital.

Our analysis of the hour of admission revealed that 93% of injuries occurred between 08:00 and 23:59, with adolescents experiencing a higher frequency of accidents during late hours (16:00–23:59) compared to the younger age groups. Furthermore, nighttime incidents (00:00–07:59) were four times more common in the adolescent group.

The peak season of injuries was between March and August (58%), particularly on weekends and in the afternoons. The mild and bright summer evenings, coupled with extended school breaks, provide ample opportunities for leisure activities, such as sports camps, excursions, and other outdoor events. These conditions also create an environment conducive to adolescents spending more time outdoors and engaging in social activities such as parties, where alcohol may be involved. This, in turn, increases the likelihood of riskier behaviors, such as riding scooters or similar vehicles, which could contribute to the higher incidence of traffic-related injuries during this period. Our results correlate with previous studies showing a higher rate of injuries during weekends and in the summer [10, 12, 23]. These findings emphasize the need for clinicians to prioritize resource allocation and preventive efforts during high-risk periods, particularly in the late hours, weekends, and summer months.

The activation of a multidisciplinary trauma team relies on protocols, prehospital evaluation of physiological and anatomical factors, and the mechanism of injury. However, in the case of pediatric injuries, additional factors outside of these protocols may lower the threshold for TTA [23].

A systematic review conducted by Drendel et al. [29] covering a span of 20 years and encompassing over 13,000 children admitted by TTA revealed that the criteria for TTA were lacking in consistency and not grounded in empirical evidence; therefore, their use frequently resulted in overtriage. Further research is needed regarding TTA to prevent unnecessary activations and inappropriate use of resources for mildly injured patients.

Another important factor influencing the decision to activate a trauma team is the hospital's setup and how the patient would otherwise be received [29]. If TTA is not triggered, the patient might be seen in the emergency department, where waiting times can be longer, despite triage systems, and where the attending physicians may have less trauma-specific experience compared to the trauma team personnel. This difference in expertise and response time could affect the initial management of pediatric trauma patients, thereby emphasizing the importance of tailored protocols for TTA.

Our study findings also suggest a tendency toward overtriage, as we see a large proportion of children with low ISS scores. This likely indicates that our threshold for TTA is too low, further emphasizing the need for more research and fine-tuning of our activation protocols to ensure optimal resource use and patient care.

## Strengths and limitations

Despite including patients over a 10-year period within a catchment area of 1.3 million people, pediatric trauma patients remain relatively rare, making it challenging to identify trends. Future studies should include nationwide populations over longer periods to analyze trends in pediatric trauma patients.

A valuable opportunity for future studies is to explore additional outcome variables beyond mortality, particularly since mortality rates are low. For children and adolescents, the loss of functional years of life is a significant factor that warrants further investigation. Thirty-day mortality was assessed using the Danish Civil Registry. It is possible that patients could have died from other causes during the 30-day follow-up period but the risk is low given that the study population consists of children.

As stated earlier in this manuscript, TTA relies on the TTA criteria, but it can be requested by the prehospital unit or emergency physicians and is consequently vulnerable to overtriage.

## Conclusions

This study highlights age-related differences in injury mechanisms, reflecting the increasing frequency of traffic-related traumas in adolescents. Although most pediatric trauma patients have mild injuries, TTA protocols must be refined to prevent overtriage and unnecessary resource utilization. The findings highlight the need for age-specific prevention strategies that focus particularly on road safety and for further research into optimizing TTA criteria.

## Data Availability

No datasets were generated or analysed during the current study.

## Appendix 1

See Table

**Table 3:** Overview of criteria for trauma team activation

Criterion	Points		
	0	1	2
Mechanism of injury	Low energy	High energy or pedestrian/bicycle	Burns (second or third degree): Children > 10% Adults > 15% Penetrating injury in head/neck
Breathing	Normal	Difficulties	Saturation < 90 Respiratory Rate < 10 or > 30
Circulation	Systolic blood pressure > 90 mmHg	Cold, pulse > 100, sweating	Systolic blood pressure < 90 mmHg
Level of consciousness	Awake	Decreased wakefulness	Unconsciousness
Thorax	No pain	Tender/pain	Open lesion
Abdomen	No pain	Tender/pain	Open lesion
Neck/spine	No pain	Tender/pain. Tingling in arms or legs	Paralysis, fractures, or suspected spinal fractures
Fractures arms, legs, pelvis	No suspicion	Extremity fractures in major long bones	Open fracture, ≥ 2 fractures in major long bones. Pelvic fracture. Amputation above the wrist or ankle
Increased risk		< 6 years > 75 years Comorbidities	
Total score:			

3.

## Abbreviations

TTA: Trauma team activation
MOI: Mechanism of injury
ISS: Injury severity score
GCS: Glasgow coma scale
AUH-TC: Aarhus University Hospital trauma center
AIS: Abbreviated injury scale
HEMS: Helicopter emergency medical service
